# Technology of Bacteria Investigation

**Published:** 1885-10

**Authors:** 


**Technology of Bacteria Investigation.** By CHARLES S. DOLLEY, M. D. Boston: S, E. Cassino. 1885.

This work is designed to interest the student in "the study of Bacteria, their culture, staining, mounting, etc., according to the methods employed by the most eminent investigators." It is a careful resume of the subject. The different plans followed by the most celebrated men, are recorded in a very short space; hence, it is useful for ready reference. This work will materially assist any beginner in the study of this most interesting subject, and be of great service to any practitioner.

